# Optimal planned missing data design for linear latent growth curve models

**DOI:** 10.3758/s13428-019-01325-y

**Published:** 2020-01-27

**Authors:** Andreas M. Brandmaier, Paolo Ghisletta, Timo von Oertzen

**Affiliations:** 1 Center for Lifespan Psychology, Max Planck Institute for Human Development, Berlin, Germany; 2 Max Planck UCL Centre for Computational Psychiatry and Ageing Research, Berlin, Germany; 3 University of Geneva, Geneva, Switzerland; 4 Faculty of Psychology, Swiss Distance Learning University, Brig, Switzerland; 5 Swiss National Center of Competences in Research LIVES - Overcoming Vulnerability: Life Course Perspectives, University of Geneva, Geneva, Switzerland; 6 Institut für Psychologie, Universität der Bundeswehr München, Neubiberg, Germany

**Keywords:** Optimal design, Random effects, Random slope, Individual differences, Power analysis, Longitudinal data

## Abstract

Longitudinal data collection is a time-consuming and cost-intensive part of developmental research. Wu et al. (2016) discussed planned missing (PM) designs that are similar in efficiency to complete designs but require fewer observations per person. The authors reported optimal PM designs for linear latent growth curve models based on extensive Monte Carlo simulations. They called for further formal investigation of the question as to how much the proposed PM mechanisms influence study design efficiency to arrive at a better understanding of PM designs. Here, we propose an approximate solution to the design problem by comparing the asymptotic effective errors of PM designs. Effective error was previously used to find optimal longitudinal study designs for complete data designs; here, we extend the approach to planned missing designs. We show how effective error is a metric for comparing the efficiency of study designs with both planned and unplanned missing data, and how earlier simulation-based results for PM designs can be explained by an asymptotic solution. Our approach is computationally more efficient than Wu et al.’s approach and leads to a better understanding of how various design factors, such as the number of measurement occasions, their temporal arrangement, attrition rates, and PM design patterns interact and how they conjointly determine design efficiency. We provide R scripts to calculate effective errors in various scenarios of PM designs.

## Introduction

Longitudinal data collection is a time-consuming, cost-intensive, and complicated part of developmental and lifespan research. The challenges include acquiring and maintaining a large participant pool, and matching participants’ time constraints, available lab space, available research assistants, and test instruments can be challenging, if not impossible. For example, developmental longitudinal neuroscientific studies exemplify these problems with the added complication a further bottleneck, the availability of the magnetic resonance tomograph (Telzer et al., 2018); but even purely behavioral studies may already become difficult to run with large numbers of participants per wave or large assessment batteries. Planned missing (PM) designs offer a principled approach to planning longitudinal studies that maximize the information that can be gained about each person, and thus ultimately, maximize statistical power while considering limited resources by strategically omitting measurement occasions for some study participants (Rhemtulla et al., 2014; McArdle, 1994; Graham et al., 2001).

Longitudinal study designs with planned missingness prescribe patterns of measurement occasions that vary across participants such that at any given measurement occasion, only a subset of all participants is measured. This is particularly interesting if testing of all participants is impossible because of monetary or physical constraints (e.g., due to limited testing facilities), or when retest or fatigue effects are to be minimized because administering the full measurement battery would be prohibitive to participants in terms of time and energy (McArdle, 1994). PM designs are derived from traditional complete designs, in which all participants are measured on all occasions, by systematically choosing only subsets of measurement occasions for subgroups of participants, and treating the remaining occasions as missing data. Because the patterns of missingness are randomly pre-assigned and thus statistically independent of both the observed and unobserved data (often referred to as a condition called Missing Completely At Random, MCAR; Rubin, 1976), they allow for unbiased parameter estimates when analyzed with any modern analysis approach that can handle missing data, e.g., structural equation models with full information maximum likelihood estimation (FIML; e.g., von Oertzen et al., 2015). The FIML approach has been compared to multiple imputation approaches, and whereas the latter generally obtain greater efficiency (i.e., smaller standard errors of parameter estimates), the two are comparable with respect to bias (i.e., recovery of correct parameter values) (Schafer and Graham, 2002).

Planning a longitudinal study involves a variety of design decisions. Typically, design decisions trade off various properties of the study (e.g., increasing participant numbers vs. decreasing cost) and optimal designs need to consider given constraints, such as total cost or a maximum total time in study for each participant. The process of planning can be regarded as a search in the space of all possible designs and thus, as an engineering task that can be solved using constrained optimization (cf. Brandmaier et al., 2015). When planning a longitudinal study, researchers need to first determine the goal of the study, which ultimately translates into maximizing precision (or ultimately statistical power) of one or more formal, statistical tests related to their hypotheses of interest. Second, researchers need to identify potential constraints and additional goals, such as the minimization of total cost or the reduction of participants’ strain. There are a variety of modifiable properties of a longitudinal study design that contribute to statistical power (Brandmaier et al., 2018b), such as the number of measurement instruments used at each occasion, their reliability, the number of measurement occasions and their temporal arrangement, the total time in study, and the total number of participants tested.

PM designs add further degrees of freedom and thus further complicate the search for an optimal design. Typical design considerations for PM designs are the number of different PM response patterns, the rule set to generate the patterns, and the proportional assignment of participants to patterns. For example, the multiform design is a simple and versatile PM design approach in which each participant is randomly presented only a subset of all available items (Graham et al., 2006). Among the many possible multiform designs, the three-form design is the most popular (Rhemtulla et al., 2014). In cross-sectional three-form design (see Table 1), all test items are divided into four sets of items (“groups”), such that one group (the “common” group; often denoted “X”) contains all items that are central to the study and will be administered to all participants. The three remaining groups (the “partial” groups; often called “A”, ”B,” and “C”) are only administered to subsets of the participants, thereby saving resources. Then, participants would either obtain items XAB, XAC, or XBC, thus saving 25% of resources per person if items are equally distributed among the groups; larger resource savings are possible when fewer items are in “X”; however, “X” considerably contributes to the efficiency of many parameters and should not be too small (Rhemtulla & Little, 2012). As a result of this standard design, only two-thirds of the participants provide answers on the items from the partial group. This idea can easily be carried over to longitudinal designs, in which we replace items with measurement occasions. Thus, a three-form longitudinal study would have all participants measured at the first wave, a partial group would be followed up on the second and third wave, another partial group would be followed up on the second and fourth wave, and a last group would be followed up on the third and fourth wave (see also McArdle & Woodcock, 1997).

**Table 1:** Example of a planned missing (PM) design. Participants are randomly assigned to each pattern. Proportion of participants in each pattern are typically identical

A three-form design				
Pattern	Test item groups			
	X	A	B	C
1	1	1	1	0
2	1	1	0	1
3	1	0	1	1

One set of items/observation is common to everybody (“X”) whereas the remaining “partial” groups are administered to subsets of participants. Whether a specific item set was observed is encoded as 1 = observed, 0 = missing

Researchers are not limited to the three-form design for longitudinal studies. Generally, there are no prior constraints to how patterns of missingness can be arranged over waves of longitudinal data collection. In principle, there is no need to have a “common” group at all and eventually, one could come up with PM designs in which each person has their unique pattern of missing data. The optimal choice of partial response patterns in PM designs depends on what statistical model one assumes and which hypotheses one deems to be of primary interest. For example, a researcher may choose between patterns with two, three, or more measurement occasions per participant, with larger numbers of occasions yielding greater statistical power for testing change-related parameters, but also at higher cost, or a researcher may choose to favor one particular pattern and attribute a larger portion of participants to it. We have previously demonstrated how *effective error* is related to reliability and statistical power (Brandmaier et al., 2018a, b), and that it can be used to develop a basis for comparing alternative research designs under identical power to test a chosen hypothesis of interest, and that this notion can be used for systematic searches in study design space (Brandmaier et al., 2015). This provides a formal way to trade off design parameters against each other without changing power or investigating the effect of the individual design decision on statistical power. Leveraging asymptotic results, there is no need to run time-intensive Monte Carlo simulations. Here, we revisit the insightful and inspiring simulation work presented by Wu et al. (2016) to investigate optimal PM designs for the study of linear change. We complement their approach, which is available as R package SEEDMC (Jia and Wu, 2015), by providing an asymptotic perspective to their empirical simulations. We aim to answer the authors’ call for the need (1) to understand “the mechanisms by which PM designs are influenced by attrition and why attrition favors one PM design over another” (Wu et al., 2016, p. 1057) and (2) to show the generalization of the efficient designs they identified for limited cases to answer broader questions about optimal design under PM.

## Method

### Latent growth curve models

To formally address the question of which PM designs are optimal for modeling longitudinal change, we follow Wu et al. (2016) and focus on latent growth curve models (LGCMs) as statistical models for change over time. LGCMs have become a commonly used analysis technique to capture change in longitudinal data (Duncan et al., 2006; Ferrer and McArdle, 2010; Meredith & Tisak, 1990). They allow for modeling average change over time, individual differences in change, and predictors of said change. In a linear LGCM, participants’ observed scores are represented by two latent factors: the intercept representing individual differences at a specified time point (often study onset), and a linear slope representing individual differences in linear change over time. Residual factors capture the otherwise unexplained variation around the growth curve, including occasion-specific variation, model misspecification, and measurement error (von Oertzen et al., 2010). In a linear LGCM, the mean vector μ, and the covariance matrix Σ of the observed variables are a function of factor loadings Λ, latent variables’ intercepts *υ*, a latent covariance matrix Ψ, and a residual covariance matrix Θ (e.g., Bollen, 1989):

1 $$ \begin{array}{@{}rcl@{}} {\Sigma} & = & {\Lambda}{\Psi}{\Lambda}^{\prime}+{\Theta} \end{array} $$

2 $$ \begin{array}{@{}rcl@{}} \mu & = & {\Lambda}\nu \end{array} $$

Under the assumption of homoscedastic and uncorrelated residual errors

3 $$ \begin{array}{@{}rcl@{}} {\Lambda} & = & \left[\begin{array}{cc} 1 & t_{1}\\ 1 & t_{2}\\ 1 & \vdots\\ 1 & t_{M} \end{array}\right] \end{array} $$

4 $$ \begin{array}{@{}rcl@{}} {\Psi} & = & \left[\begin{array}{cc} {\sigma_{I}^{2}} & \sigma_{IS}\\ \sigma_{IS} & {\sigma_{S}^{2}} \end{array}\right] \end{array} $$

5 $$ \begin{array}{@{}rcl@{}} \nu & = & \left[\begin{array}{c} \mu_{I}\\ \mu_{S} \end{array}\right] \end{array} $$

6 $$ \begin{array}{@{}rcl@{}} {\Theta} & = & \left[\begin{array}{ccc} \sigma_{\epsilon}^{2} & 0 & 0\\ 0 & {\ddots} & 0\\ 0 & 0 & \sigma_{\epsilon}^{2} \end{array}\right] \end{array} $$

with the number of measurement occasions *M*, at times *t*_1_ to *t*_*M*_, the residual error $\sigma _{\epsilon }^{2}$, the mean *μ*_*I*_, and variance of the latent intercept ${\sigma _{I}^{2}}$, and the mean *μ*_*S*_, and the variance of the latent slope ${\sigma _{S}^{2}}$, and the latent intercept-slope-covariance *σ*_*IS*_. The loadings *t*_1_ to *t*_*M*_ are fixed and typically, the intercept is anchored at the first measurement time point (even though other choices are possible) *t*_1_ = 0, and *t*_*M*_ represents a quantity proportional to the time elapsed since the beginning of the study.

### Effective error for complete data

To estimate the precision with which structural equation models measure latent constructs of interest, such as the intercept or the slope in linear LGCMs, von Oertzen (2010), von Oertzen & Brandmaier (2013) developed the concept of “effective error” based on the idea of power equivalence. Two models are power-equivalent if they have identical power to detect a given effect of interest. For example, a LGCM with seven measurement occasions over 12 years may be power-equivalent to a LGCM with four measurement occasions over fourteen years (the exact trade-off depends on further assumptions such as indicator reliability and that the model holds over both timespans; von Oertzen & Brandmaier, 2013). Using power-equivalent transformations, one can derive a hypothetical, minimal model that directly measures a latent construct of interest, e.g., the latent slope with a single residual error term. Then, the effective error is the measurement error for this hypothetical direct measurement of the latent variable under investigation. For example, if we are interested in a hypothesis test on the linear slope in a LGCM, effective error is the measurement error we would face if we had measured the linear slope directly. We could imagine the complete study with its multiple occasions of measurements as a single measurement instrument that outputs only a single value (the linear slope estimate) for each person. Then, the effective error would be the measurement error of that entire study for measuring the linear slope. For Wald-type tests on complete designs, Brandmaier et al. (2018b) have shown that the effective error for measuring the slope variance in an LGCM is

7 $$ \sigma_{eff}^{2}=\frac{\sigma_{\epsilon}^{2}}{{\sum}_{j=1}^{M}{t_{j}^{2}}-\frac{1}{M}\left( {\sum}_{j=1}^{M}t_{j}\right)^{2}} $$

The main benefit of the effective error metric is that it is independent of sample size, test size (also referred to as alpha level) and true effect size. Brandmaier et al. (2018b) have shown that effective error can be differently rescaled to obtain other measures of sensitivity to detect effects of interest, such as reliability (by scaling with true effect size) or power (by scaling with true effect size, sample size, and test size). Others have investigated LGCM with respect to their *efficiency*, which is typically defined as the squared standard error for a parameter (Rhemtulla and Little, 2012; Wu et al., 2016). Then, the ratio of two models’ efficiencies is of particular interest as a measure of relative efficiency. This line of thinking is closely connected to effective error. We can rescale effective error with sample size to obtain an asymptotic estimate of the standard error of the parameter of interest, which we term *effective standard error.* For example, the effective standard error of the slope variance ${\sigma _{S}^{2}}$ in a linear LGCM with effective error $\sigma _{eff}^{2}$ and sample size *N* (cf. Ahn & Fessler 2003) is

8 $$ se_{eff}=\sqrt{\frac{2}{N-1}}\left( {\sigma_{S}^{2}}+\sigma_{eff}^{2}\right) $$

Both effective error and efficiency are variance metrics and thus, we can similarly use the ratio of two effective errors to compare relative efficiency of designs. Rhemtulla and Little (2012) and Wu et al. (2016) have suggested that such relative efficiencies can also be translated into width inflation factors, which reflect the extent to which a confidence interval around the parameter of interest is expected to be inflated.

The idea of effective error was extended to optimization in study design space by Brandmaier et al. (2015), who proposed to systematically evaluate the efficiency of alternative longitudinal study designs by means of comparing their effective errors. In the remainder of this paper, we will extend their approach to gauge efficiency of PM data designs. This approach allows us to obtain asymptotic estimates of precision and reliability that can be leveraged to better understand how design features relate to precision, to make final design decisions, or to narrow down a small candidate pool of designs that is then further explored using simulation-based approaches. These yield unbiased estimates of precision and power and allow further inquiry of properties like non-convergence rates.

### Optimal design when studying linear change

**Fig. 1:**
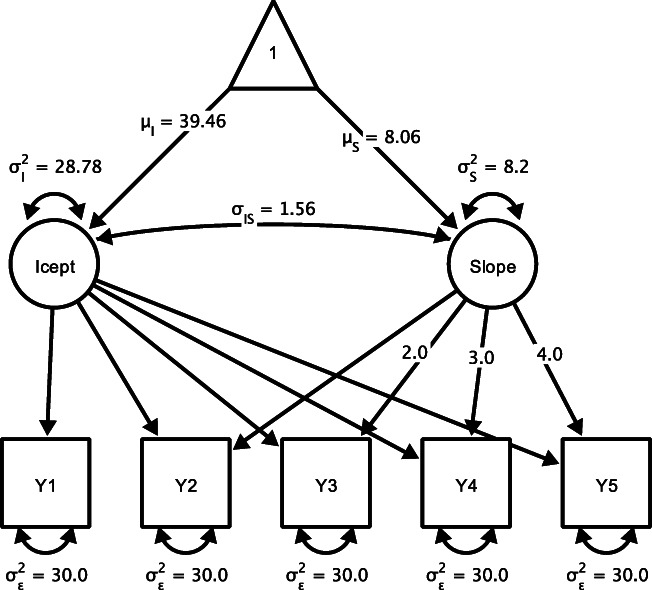
Latent growth curve model with five measurement occasions. The parametrization shown corresponds to the reference model considered throughout. Icept = Intercept

In their first simulation, Wu et al. (2016) considered efficiency to test parameters of a linear latent growth curve model. For a given sample size, they determined total sample size *N* = *B*/(*T* ⋅ *C*) with a total budget *B*, the cost of collecting a single data point *C*, and the number of repeated measures per pattern *T* assuming that the collection of every data point incurs the same cost. In a Monte Carlo simulation, the authors evaluated empirical sampling variances, that is, the variance of the Monte Carlo parameter estimates across repetitions. To compare alternative designs, the authors reported relative efficiency, that is, the ratio of the efficiency of a given design and the efficiency of the most efficient design. In their example, the budget was $100,000 and each assessment cost $20, which yields a sample size of 1,666 participants for designs with three occasions or of 1,250 participants for designs with four occasions. Population parameters of the linear model reported in Wu et al. (2016) were adapted from a study by Biesanz et al. (2004) and were as follows: The latent intercept has a mean *μ*_*I*_ = 39.46 and variance ${\sigma _{I}^{2}}=28.78$, the latent slope had a mean *μ*_*S*_ = 8.06, and variance ${\sigma _{S}^{2}}=8.20$, and the covariance of the intercept and the slope was $\sigma _{IS}^{2}=1.56$, equaling a correlation of *r* = 1.10. Residual error variances were constrained to be equal across time points and set to $\sigma _{\epsilon }^{2}=30$. The five occasions of measurement were equidistant. The corresponding path diagram is shown in Fig. 1.

Wu et al. (2016) considered either complete designs (CD) or PM designs. They defined CDs as those designs in which all participants are assessed at the same time points (even though not all possible time points have to be in a CD). Henceforth, we will use their notation to refer to CD and PM designs. A PM study is given in curly brackets containing all possible PM response patterns separated by vertical bars. Each response pattern indicates which measurement occasions were observed. For example, the aforementioned three-form design (see Table 1) could be written as {1,2,3 | 1,2,4 | 1,3,4}. Unless otherwise noted, we assume that participants are randomly and in equal shares distributed to the response patterns. So, in this example, one-third of the participants were measured at the first, second, and third time point (that is, in pattern {1,2,3}), another third at the first, second, and fourth time point (that is, {1,2,4}), and the last third at the first, third, and fourth time point (that is, {1,3,4}).

First, we will investigate the efficiency of all possible CDs that will later be the set of partial response patterns from which we can select and that we can then combine to generate PM designs. All possible CDs with three out of five measurement time points and their asymptotic effective errors are listed in Table 2. Holding all design parameters constant but varying only the arrangement of the measurement occasions in time, we see from Eq. 7 that the designs that maximize efficiency are those with the largest variance in the time points (Var(t)). This translates to a simple rule-of-thumb for the design of longitudinal studies under a linear model of change: Measure as often as possible very early in the study and measure as often as possible towards its end. Asymptotically, this strategy reduces the optimal design to a two-point latent change score model with optimal (multiple-)indicator^1^ reliability (see Kievit et al., 2018; Willett, 1989). This heuristic conforms with the finding by Wu et al. (2016) that among the models restricted to any 3 of the 5 possible measurement occasions, the designs {1,2,5} and {1,4,5} were the most efficient; while the one with equally spaced measurement occasions, {1,3,5}, was not optimal. Both designs {1,2,5} and {1,4,5} maximize the variance of measurement points in time among the available designs (see Table 2 for all possible designs and their variances). It follows from our formal derivation that this finding is asymptotically true irrespective of the other parameter values of the LGCM, which are held constant across alternative designs. Furthermore, as von Oertzen and Brandmaier (2013) already noted, it is typically beneficial to prolong the total study time span to maximize statistical power for finding effects if there is no attrition. However, in realistic settings, increasing attrition over time will counteract the beneficial effect of waiting for slope-related individual differences to get larger over time.

**Table 2:** All possible complete designs with three out of five equidistant measurement occasions (Design), the variance of the time points (Var(t)), and their effective error for a Wald test of slope variance (EE; effective error)

Design	Var(t)	EE	Design	Var(t)	EE
{1,2,3}	1	15	{1,4,5}	4.33 (*)	3.46
{1,2,4}	2.33	6.43	{2,3,4}	1	15
{1,2,5}	4.33 (*)	3.46	{2,3,5}	2.33	6.43
{1,3,4}	2.33	6.43	{2,4,5}	2.33	6.43
{1,3,5}	4	3.75	{3,4,5}	1	15

Designs with optimal variance are denoted with an *asterisk*. Results are displayed in two adjacent columns

### Effective error for planned missingness designs

**Fig. 2:**
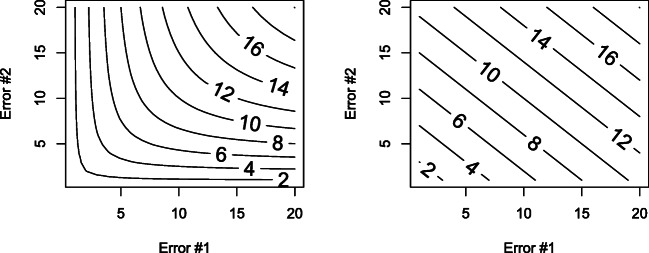
Harmonic (*left*) and arithmetic (*right*) mean of effective errors. Effective errors (represented as Error #1 and Error #2) combine according to the harmonic mean (left)

How do the previous considerations translate and generalize to PM designs? Without attrition, Wu et al. (2016) report {1,5 | 2,5 | 3,5 | 4,5} as the most efficient PM design with five measurement occasions. To fully understand the effects that contribute to optimal efficiency, we extend effective error for CDs as introduced in Eq. 7 to effective error for PM designs. von Oertzen and Brandmaier (2013) derived the effective error for study designs including multiple independent groups. They found that the total effective error of the design is the sample-size-weighted harmonic mean of the group-specific effective errors (adapted from Theorem 4 in the Appendix of von Oertzen and Brandmaier (2013); see also Dolan et al., 2004):

9 $$ \sigma_{eff}^{2}=\frac{N}{{\sum}_{j=1}^{k}\frac{Nj}{\sigma_{eff,j}^{2}}} $$

with *k* being the number of groups with group *j* having sample size *N*_*j*_ and effective error $\sigma _{eff,j}^{2}$. That is, if half of the participants have an effective error of *e*_1_ and the other half have an effective error of *e*_2_, the effective error of the design is $\frac {1}{\frac {1}{e_{1}}+\frac {1}{e_{2}}}=\frac {e_{1}e_{2}}{e_{1}+e_{2}}$ (Fig. 2). PM designs can be thought of as designs with multiple independent groups with each group corresponding to a different partial response pattern. Wu et al. (2016) assumed only designs with equal proportions of participants, so we can simplify Eq. 9 (ignoring the absolute scale of $\sigma _{eff}^{2}$) to

10 $$ \sigma_{eff}^{2}\propto\frac{j}{{\sum}_{j=1}^{k}\frac{1}{\sigma_{eff,j}^{2}}} $$

when comparing alternative study designs with identical sample size. For example, if we wanted to compute the effective error of a three-form design {1,3,5 | 1,2,5 | 1,4,5} and we knew that their respective effective errors were 3.75, 3.46, and 3.46 (see Table 2), we would obtain an effective error of

11 $$ \sigma_{eff}^{2}=\frac{3}{1/3.75+1/3.46+1/3.46}=3.55 $$

We conclude that we need to choose the elements of a PM such that a value proportional to the harmonic mean of the effective errors is maximized. The harmonic mean is dominated by the minimum of its arguments:

12 $$ \min\left( \sigma_{eff,1}^{2},\ldots,\sigma_{eff,j}^{2}\right)\!\leq\!\sigma_{eff}^{2}\!\leq\! j\cdot\min\left( \sigma_{eff,1}^{2},\ldots,\sigma_{eff,j}^{2}\right) $$

In other words, the effective error of a PM design cannot be lower than the effective error of any single partial response pattern, and the effective error cannot increase without limits when we decrease the precision of any pattern but the pattern with the minimum effective error.

By means of Monte Carlo simulation, Wu et al. (2016) examined alternative designs constituting combinations of the response patterns shown in Table 2, that is, combinations of response patterns that always included the first and last wave for each participant. Table 3 lists the ten most efficient designs for detecting slope variance including the original simulation results and our asymptotic results. The left hand side of the table (caption: SEEDMC) repeats Monte Carlo simulated results from Wu et al. (2016), and their relative efficiency to the best design ({1,2,5}). The right hand side of the table shows asymptotic results based on effective error. In the original paper, the designs were derived under a fixed cost constraint of 5,000 monetary units, where inviting a participant to one occasion costs 1 unit. With three measurement occasions (e.g., {1,2,5}), we could afford 5,000/3 = 1,666 participants, and with four occasions, 5,000/4 = 1,250. Thus, we had to adjust the effective errors for the different sample sizes. We did so by computing a discounting factor based on the unequal sample sizes as *ψ* = 1,666/1,250 = 1.34. This discounting factor is used to re-scale the effective errors of all studies with 1,250 participants, such that we obtain a sample-size adjusted metric for effective errors. From Table 3, we find that the Monte Carlo simulation and our asymptotic approach produced the identical rank order of optimal designs. At the same time, there are small discrepancies due to the Monte Carlo estimation procedure such that some designs appear to show marginal differences when they are actually identical (e.g., C4 and C6), whereas others appear identical although they are really slightly different (e.g., M45 and M23).

**Table 3:** Top ten designs for the variance parameter of the slope in a linear growth curve model without attrition

SEEDMC					Asymptotic approach		
Rank	Design	*N*	Response pattern	RE	EE	AEE	RE
1	C1	1,666	{1,2,5}	1.00	3.46	3.46	1.00
2	C3	1,666	{1,4,5}	0.98	3.46	3.46	1.00
3	M33	1,666	{1,2,5 | 1,3,5 | 1,4,5}	0.97	3.55	3.55	0.97
4	C2	1,666	{1,3,5}	0.94	3.75	3.75	0.92
5	C5	1,250	{1,2,4,5}	0.85	3.00	4.00	0.86
6	M42	1,250	{1,2,3,5 | 1,2,4,5}	0.82	3.2	4.26	0.81
7	M45	1,250	{1,2,4,5 | 1,3,4,5}	0.81	3.2	4.26	0.81
8	M23	1,250	{1,2,3,5 | 1,2,4,5 | 1,3,4,5}	0.81	3.27	4.36	0.79
9	C6	1,250	{1,3,4,5}	0.79	3.43	4.57	0.76
10	C4	1,250	{1,2,3,5}	0.78	3.43	4.57	0.76

The order of designs corresponds to the order reported in the supplementary materials of the Wu et al. (2016) simulation #4; p. 2. Columns show the rank order (rank), design name (design), the sample size feasible under the pre-specified cost constraint (*N*), the pattern of observations (response pattern), efficiency relative to the best model according to the simulation by Wu et al. (2016) (RE; relative efficiency). The final three columns show the result of our approach, effective error variance (EE; effective error), adjusted effective error (AEE), and relative efficiency (RE) as the ratio of a design’s AEE and the best AEE

With an asymptotic approach, it is straightforward to expand the range of possible design considerations beyond what is practically feasible using a Monte Carlo approach. For example, we could derive all effective errors for all missing response patterns of interest (see Table 4) in a single run, and then search for arbitrary combinations of them. These combinations could include designs in which response patterns with different numbers of measurement occasions are mixed, e.g., {1,2 | 1,2,3 | 1,2,3,4 | 1,2,3,4,5}, or those in which the number of participants is unequal across response patterns. For evaluating combinations of mixed patterns, one first estimates effective error of each pattern using Eq. 7. Then, one simply applies the combination rule from Eq. 9, to arrive at the final effective error. In the example just given, we would obtain the following effective errors: 60 (for pattern {1,2}), 15 (for pattern {1,2,3}), six (for pattern {1,2,3,4}), and three (for pattern {1,2,3,4,5}). Using the combination rule, we obtain an effective error of 6.86 when assigning participants equally to the five patterns. The combination rule easily allows us to assign different proportions of participants (*N*_*j*_ for pattern *j* in Eq. 9) to each pattern. If we increased the proportion of participants in the most effective pattern ({1,2,3,4,5}) to 70% and assigned each 10% to the remaining patterns, we obtain an effective error of 3.87 whereas if we assigned 70% to the least effective pattern ({1,2}) and 10% to the remaining patterns, we obtain an effective error of 14.63. We provide R code (https://osf.io/ezgq3/) to reproduce these computations.

**Table 4:** Effective errors for missing patterns derived from pattern C7 {1,2,3,4,5} that keep the first and last occasion of measurement

Design	Pattern	N	Effective error	Effective SE	Relative error
C7	{1,2,3,4,5}	1,000	3	0.50	1.0
C5	{1,2,4,5}	1,250	3	0.45	1.0
C6	{1,3,4,5}	1,250	3.43	0.47	0.88
C4	{1,2,3,5}	1,250	3.43	0.47	0.88
C1	{1,2,5}	1,666	3.46	0.40	0.87
C3	{1,4,5}	1,666	3.46	0.40	0.87
C2	{1,3,5}	1,666	3.75	0.41	0.80

Columns describe the pattern name (design), the pattern of measurement occasions (pattern), sample size (*N*), effective error and effective standard error (effective SE), and the relative error as the ratio of effective errors

Note that an initial analysis of the potential partial response patterns may be insightful even before selecting and combining patterns into potential study designs. For example, removing the centered occasion (measurement at time point 3) from C7 (resulting in design C5) has virtually no influence on the power to detect individual differences in change (see Table 4, and our earlier discussion on the importance of estimating the end points optimally) but allows an increase in sample size by 25%, resulting in a much more efficient design. von Oertzen and Brandmaier (2013) have empirically demonstrated a similar effect using six-wave longitudinal data gathered over 13 years in the Berlin Aging Study, an interdisciplinary and longitudinal study of very old individuals (Baltes & Mayer, 1999); for a selected cognitive task (retrieval of semantic categories from long-term memory), they showed that the statistical power for hypotheses on the linear slope remained nearly identical when removing an entire longitudinal wave^2^. Of course, all these considerations only hold true assuming that the linear change model is correctly specified, and in general, researchers may need to pay respect to other design features and goals when selecting a final study design.

### Effective error for unplanned missingness designs

Our earlier considerations assumed PM designs without attrition, that is, all missingness was assumed to be planned a priori. However, longitudinal studies will almost always include unplanned missing data. In order to model random participant drop-out, Wu et al. (2016) considered three attrition groups in their simulation, with either no, low, or high attrition. Attrition was assumed to be a linear function of time and the probabilistic missing data mechanism was formalized as a probability of missing data for person *i* at occasion *j* that linearly depends on time elapsed and was independent of the observed variables:

$$ p\left( y_{ij} \mathrm{is\ missing}\right)=\upsilon\cdot t_{j} $$

with *υ* representing the linear attrition rate per unit of time, and *t*_*i*_ the time elapsed between study onset and the *i*-th occasion. They chose *υ* =.075 (low attrition), and *υ* =.175 (high attrition), which amounts to the proportion of participants in the study not returning per unit of time.

Wu et al. (2016) noted that, with greater attrition, SEEDMC tends to select those designs including later rather than earlier time points. This may initially seem counterintuitive because measurements at later time points become less efficient due to the higher proportion of drop-outs. For example whereas the designs {1,2,5} and {1,4,5} are equally efficient under no attrition (same variance, same effective error), {1,4,5} is superior to {1,2,5} under mild attrition, and even more so under high attrition. To better understand this effect, we can formalize it in our framework by leveraging the combination rule for effective error from multiple groups as shown in Eq. 9:

13 $$ \sigma_{eff}^{2}=\frac{N}{{\sum}_{j=1}^{k}\frac{N_{j}}{\sigma_{eff,j}^{2}}} $$

with $N_{j}=N\cdot \upsilon \cdot \left (t_{j}-1\right )$ considering *k* groups of distinct patterns of missing data. Note that we can treat a CD with missing data as a PM design, the only difference being that the cause of missingness is unplanned. For the analysis and derivations of error, this distinction is irrelevant under MCAR assumptions. Effectively, a {1,2,5} design with random attrition is a {1 | 1,2 | 1,2,5} design, in which some people drop out after the first occasion {1}, some after the second {1,2}, and some perform all three measurement occasions {1,2,5}. In standard wave missing design, the proportions of persons in every response pattern is equal. With unplanned missingness, the attrition rate determines the proportions of persons in every response pattern. To formalize this, we propose the following extended notation based on the original notation of Wu et al. (2016), in which we denote the response patterns (as before) and in addition, the proportion of cases in each pattern separated by a colon. For example, a PM design in which the response patterns always include two of all four measurement occasions, always have the first measurement occasion, and participants are evenly distributed among the partial response patterns is written as {1,2 : 25% | 1,3 : 25%, 1,4 : 25% | 1,5 : 25%}. This allows us to denote implied missingness designs by considering attrition rates. For example, we can derive the implied response patterns from the original response patterns {1,2,5} and {1,4,5} given low and high attrition. The proportion of missing persons at the *j* th measurement can be calculated as the sum of all persons that are still in the study at the *j*-th measurement minus all persons that are measured in response patterns with measurements up to the $\left (j-1\right )^{th}$ occasion, that is, we obtain the implied designs {1 : 7.5% | 1,2 : 22.5% | 1,2,5 : 70% } and {1: 22.5% | 1,4 : 7.5% | 1,4,5 : 70%} for the low attrition condition (*υ* = .075).

The participants with block {1} do not directly contribute to the precision of estimation of slope effects because change cannot be observed on the basis of a single observation. Rather, the participants with a single measurement contribute to the precision of other parameters, such as intercept variance and residual error variance. As shown in Table 4, the respective last patterns in each pattern {1,2,5} and {1,4,5} are equally efficient (in terms of their variance contribution to effective error). They are represented with equal proportions (70%) in both response patterns. Thus, the relative efficiency of both designs is guided by the question whether having to wait twice as long (going from {1,2} to {1,4}) can compensate for the fact that sample size is approximately reduced to a third (7.5%/22.5% $=\frac {1}{3}$) of the original number of participants by then. The effective error for {1,2} is $\sigma _{eff\{1,2\}}^{2}=14.7$ and for {1,4}, $\sigma _{eff\{1,4\}}^{2}=3.4$. The effective error is lower by a factor of more than 4 ($\sigma _{eff\{1,2\}}^{2}/\sigma _{eff\{1,4\}}^{2}=4.3$) when waiting for twice the amount of time. This outweighs the fact that this more precise measurement of the linear slope is only performed with a third of the participants.

Again, we investigated to what extent the asymptotic approach was able to reflect the rank order structure of optimal designs reported by Wu et al. (2016). The results are shown in Table 5. It shows the four most efficient designs for low and high attrition rates respectively, their response patterns, and total sample size as originally reported. In addition, we report effective error and sample-size adjusted effective error, which is then used to compute relative efficiency to the adjusted effective error of the most efficient design. The asymptotic approach is able to identify the most efficient design as the one with the lowest adjusted effective error and reflects the same rank order as the Monte Carlo simulation. From this, we conclude that the asymptotic approach provides a useful and computationally cheap alternative for deriving optimal designs under both planned and unplanned missing data.

**Table 5:** The four most efficient designs for hypotheses on the slope variance in linear latent growth curve models with low and high attrition rates

Attrition	Rank	Design	Pattern	*N*	EE	AEE	RE
Low	1	C3	{1,4,5}	1,666	4.68	4.68	1.0
	2	M33	{1,2,5 | 1,3,5 | 1,4,5}	1,666	4.87	4.87	0.96
	3	C1	{1,2,5}	1,666	4.85	4.85	0.96
	4	C5	{1,2,4,5}	1,250	4.04	5.39	0.87
High	1	C5	{1,2,4,5}	1,250	7.52	10.02	1.0
	2	M45	{1,2,4,5 | 1,3,4,5}	1,250	7.71	10.28	0.97
	3	C6	{1,3,4,5}	1,250	7.91	10.55	0.95
	4	M29	{1,2,4,5 | 1,3,4,5 | 2,3,4,5}	1,250	9.26	12.35	0.81

Columns show level of attrition rate (attrition), the rank order of the designs (rank), name of the design (design), pattern of missing data (pattern), the affordable sample size under the chosen cost constraint (*N*), effective error (EE), adjusted effective error (AEE), and relative efficiency based on the ratio of a model’s AEE and the optimal AEE

### Effective error for hypotheses on intercept variance

Cost effectiveness or design efficiency of a study with respect to the slope component of a linear latent growth curve may not be the only criterion for optimal design. One may also be interested in detecting reliable differences at the intercept level, that is, differences in general level of performance. To this end, we derived effective error for the intercept component in a linear LGCM. Following the same principles as described in the Appendix of Brandmaier et al. (2018b), we obtain the corresponding effective error:

14 $$ \sigma_{eff}^{2}=\frac{\sigma_{\epsilon}^{2}}{M-\eta\left( {\sum}_{j=1}^{M}t_{j}\right)^{2}} $$

with $\eta =\frac {1}{{\sum }_{j=1}^{M}{t_{j}^{2}}+\frac {{\sigma _{e}^{2}}}{{\sigma _{S}^{2}}}}$. Following the derivations of Brandmaier et al. (2018b), we obtain the limiting case as $\eta =\frac {1}{{\sum }_{j=1}^{M}{t_{j}^{2}}}$ and thus, the effective error for Wald-type tests is:

15 $$ \sigma_{eff}^{2}=\frac{\sigma_{\epsilon}^{2}}{M-\frac{\left( {\sum}_{j=1}^{M}t_{j}\right)^{2}}{\left( {\sum}_{j=1}^{M}{t_{j}^{2}}\right)}} $$

From this, we can infer that, asymptotically, there is no effect of total time on the precision with which we can measure intercept variance. Assume a factor *λ* with which we prolong the total study time span, such that $\tilde {t_{j}}=\lambda t_{j}$, then effective error remains constant for any *λ* > 0:

16 $$ \begin{array}{@{}rcl@{}} \frac{\left( {\sum}_{j=1}^{M}\lambda t_{j}\right)^{2}}{{\sum}_{j=1}^{M}\left( \lambda t_{j}\right)^{2}}&=&\frac{\left( \lambda{\sum}_{j=1}^{M}t_{j}\right)^{2}}{{\sum}_{j=1}^{M}\lambda^{2}{t_{j}^{2}}}=\frac{\lambda^{2}\left( {\sum}_{j=1}^{M}t_{j}\right)^{2}}{\lambda^{2}\left( {\sum}_{j=1}^{M}{t_{j}^{2}}\right)}\\&=&\frac{\left( {\sum}_{j=1}^{M}t_{j}\right)^{2}}{{\sum}_{j=1}^{M}{t_{j}^{2}}} \end{array} $$

Table 6 shows optimal designs to detect variance of intercept as reported by Wu et al. (2016). It parallels Table 3 by showing both the Monte Carlo estimates derived from SEEDMC and our asymptotical findings using the effective error for intercept variance. We find that the asymptotic results largely reflect the Monte Carlo findings. The rank order of results is identical with the exception of designs M42, M10, M33, and C2, which yielded overoptimistic (that is, too small) effective error values. Still, the asymptotic approach proves valuable as a simple and computationally cheap alternative to Monte Carlo simulations (Table 7).

**Table 6:** Top ten designs (and designs C2, C3) for the variance parameter of the intercept in a linear growth curve model without attrition

SEEDMC				Asymptotic approach			
Rank	Design	N	Response pattern	RE	EE	AEE	RE
1	C1	1666	{1,2,5}	1.0	19.62	19.62	1.0
2	M30	1666	{1,2,3 | 1,2,4 | 1,2,5}	0.95	21.79	21.79	0.90
3	C4	1250	{1,2,3,5}	0.89	18.00	24.00	0.82
4	M42	1250	{1,2,3,5 | 1,2,4,5}	0.85	18.72	24.96	0.79
5	M10	1666	{1,2,3 | 1,2,4 | 1,2,5 | 1,3,4 | 1,3,5 | 1,4,5}	0.84	24.17	24.17	0.81
6	M40	1250	{1,2,3,4 | 1,2,3,5}	0.83	19.38	25.85	0.76
7	M20	1250	{1,2,3,4 | 1,2,3,5 | 1,2,4,5}	0.82	19.42	25.90	0.76
8	M33	1666	{1,2,5 | 1,3,5 | 1,4,5}	0.82	23.88	23.88	0.82
9	C5	1250	{1,2,4,5}	0.81	19.50	26.00	0.75
10	M41	1250	{1,2,3,4 | 1,2,4,5}	0.79	20.22	26.96	0.73
12	C2	1666	{1,3,5}	0.79	25.00	25.00	0.78
29	C3	1666	{1,4,5}	0.65	28.85	28.85	0.68

The order of designs corresponds to the order reported in the supplementary materials of Wu et al. (2016), simulation #3; p. 2. Columns show the rank order (rank), design name (design), the sample size feasible under the pre-specified cost constraint (*N*), the pattern of observations (response pattern), efficiency relative to the best model according to the simulation by Wu et al. (2016) (RE; relative efficiency). The last three columns show the result of our approach: effective error (EE); adjusted effective error (AEE), and relative efficiency (RE) the ratio of a design’s AEE and the best AEE.

**Table 7:** Monte Carlo simulated effective standard errors for intercept variance listed for all complete designs with three or four measurement occasions

Design	EE	Design	EE	Design	EE
{1,2,3}	2.05	{1,4,5}	2.23	{2,3,4,5}	3.21
{1,2,4}	1.89	{2,3,4}	4.18	{1,3,4,5}	2.26
{1,2,5}	1.81	{2,3,5}	2.98	{1,2,4,5}	2.01
{1,3,4}	2.18	{2,4,5}	3.49	{1,2,3,5}	1.93
{1,3,5}	2.05	{3,4,5}	7.77	{1,2,3,4}	2.08

Columns show the design pattern (design) and their effective errors (EE). Results are displayed in three adjacent columns

### Effective error for mean parameters

So far, we have focused on the effective error for variance parameters. In practice, mean-related parameters are tested as well, for example, when researchers investigate average change, or if there is a mean effect of an exogeneous variable on the latent variable of interest. Here, we briefly discuss that our approach can be used to study these settings as well. Asymptotically, the effective error is the inverse of the precision with which we can measure a latent variable (Brandmaier et al., 2018b). Thus, the effective error applies identically to both hypotheses about the mean or the variance of a latent variable of interest. In other words, any change to a study design that increases the precision of the slope variance asymptotically also increases the precision of the slope mean. The prediction of our approach is simply that, under the asymptotic assumptions, the rank order of optimal designs is identical for tests on means and variances. The prediction that both the effect of exogenous predictors and tests on the means produce the same rank order are confirmed by Wu et al. (2016, see their Supplementary Materials). For example, under no attrition, the most efficient designs for tests on the mean slope are reported as M18, C3, C1 (their Table 4) and the same rank order is reported for tests on the effect of an exogeneous predictor. Note that we have identified C1 and C3 as the best designs with almost identical efficiency but not M18 (see our Table 3), which is defined as {1,5 | 2,5 | 3,5 | 4,5}. We discuss the implications of this difference between asymptotic and simulation-based results in the Limitations section below.

## Discussion

Longitudinal data collection is a resource-costly and time-intensive part of research focusing on within-person change, for instance, when investigating questions of child development and aging. Planned missing data designs provide an excellent opportunity to maximize study design efficiency (McArdle, 1994). Recent advances in computational power endow researchers with means to simulate a variety of alternative designs such that a design with optimal power or optimal resource investment can be selected. Wu et al. (2016) have made an important contribution to study design planning by extending considerations on optimal design from CDs to PM designs. In this article, we extend their important work and provide firm grounds for efficiently planning study designs by providing an asymptotic solution to the underlying design problem that is computationally much more efficient than a simulation-based approach. As we have noted earlier, Monte Carlo simulations can be time-consuming, particularly when large spaces of possible study designs need to be searched. Even though parallelization of computations can mitigate the problem to some degree, the exponential growth of the problem space will always limit the practicality of the approach (in terms of computation time) when the degrees of freedom of the design are large. In addition, the current implementation of SEEDMC relies on Mplus (Muthén and Muthén, 2007), a closed-source commercial application that may not be available to all researchers. Finally, an asymptotic solution allows us to fully examine and understand underlying principles about how design facets relate to statistical power. Here, we have specifically revisited alternative PM designs for the study of effects on the linear slope in a LGCM from the perspective of effective error, reliability, and statistical power (also see Brandmaier et al., 2015, 2018b).

As we mentioned earlier, a comprehensive understanding of how specific facets of a study design influence statistical power frees researchers from unnecessary constraints in designing future studies. There is less of a need for basing future studies on existing study designs, which may unnecessarily restrict researchers’ exploration of even more efficient designs. Also, there is less of a need to base future studies on simple heuristics. Specifically, we disagree with the heuristics given by Wu et al. (2016) that, for a linear change process, the optimal allocation of repeated measures in a CD design is approximately equally spaced. With Eq. 7, we can show that this is not true under the assumptions of a standard LGCM (cf. Willett, 1989). In fact, the design that maximizes the variance over the measurement time points yields optimal power. Variance is maximal if we place half of the measurements on the first occasion, i.e., repeat the measurements within a short time on the first day of the study, and the remaining half on the second and last occasion, again repeating the measurements within a short time on the last day of the study. Of course, this is assuming independence of the measurements, that is, no spill-over or retest effects from repeated assessments on a single day. Wu et al. (2016) based their conclusion on findings from Tekle et al. (2011) who considered designs with an auto-regressive structure and showed that equal spacing is more efficient the larger the auto-regressive coefficient is; without an auto-regressive component in the model, however, this claim no longer holds. In other cases (specifically, see bottom left panel of their Fig. 2; and likewise the panels of Fig. 1 in Ouwens et al. 2002, that shows the same picture for when the auto-correlation approaches zero), the distributions of time points that maximize the variance are optimal.

### Limitations

As with any approach of statistical power analysis, a priori values for all model parameters must be assumed. If no values are known, researchers must aim at providing conservative values, such as low indicator reliability and small effect sizes. Any uncertainty or bias in the a priori values is reflected in uncertainty or bias in the estimates of effective error and/or relative efficiency; similarly, misspecification of the model may lead to deviation from the optimal solution; however, this is true for both simulation-based and asymptotic approaches and a general issue in optimal study design.

In practice, every now and then models do not converge to an admissible solution. Reasons for non-convergence are various and one such reason is empirical underidentification, which may arise due to low sample size. Our asymptotic approach is over-optimistic in the sense that it operates under the assumption that non-convergence rate is unchanged in expectation by the power-equivalent transformations. If non-convergence rate is an issue or of specific interest, one needs to resort to simulation-based approaches.

Our current approach is limited to models of linear change with independently and identically distributed residual errors. These models are typically used when the modeled time span is relatively short and when the direction of change rather than the exact shape or the temporal dynamics are of interest (cf. Ghisletta et al., 2020). Rhemtulla and Little (2012) implemented a more general asymptotic approach to computing efficiency for MCAR designs. Their approach does not have the benefit of producing an elegant formula that can be inspected (e.g., to see that effective error decreases as variance of the time points increases) but it has the advantage that it can be applied to any model (such as models of quadratic change) and any pattern of MCAR data. One must keep in mind that, no matter what approach is taken, optimizing designs for linear change may decrease their ability to detect non-linear change. Also, note that the linear models discussed here and in Wu et al. (2016) do not model retest effects, that is, we assume that the change in the dependent variable of interest is completely described by the time relation as in Eq. 3, and not subject to additional influences directly related to number of previous exposures to that variable. In principle, it is possible to consider explicit models of retest effects in the considerations presented here but an implementation of this idea remains as future work.

Simulation approaches are generally more flexible with respect to extensions to other types of non-linear growth curves, other estimators, non-normality of errors, or other types of inference procedures (e.g., weighted-least-squares-based estimation). At the same time, they often impose limitations for computational reasons. For instance, Wu et al. (2016) limited the set of possible designs to reduce the search space to a practical searchable space. Indeed, they reduced the space of possible CDs to only those models with three out of five measurement occasions. However, optimal PM designs may include subgroups that are only tested on any subset of measurement occasions, such as only the last two or only the first two. In particular, the search space typically grows exponentially with the number of measurement occasions. An asymptotic approach is much more flexible with respect to the optimization of a study design in a well-defined search space as it does not require computationally expensive Monte Carlo simulation but offers an optimal solution under formal models of change considering both planned and non-planned attrition. Still, one must keep in mind that the asymptotic assumptions may be violated with small to moderate sample sizes and optimal design solutions are rather approximate than exact. However, approximate solutions help us build a general understanding of how design decisions trade off against each other and how design parameters affect precision and power.

Last, we would like to note that the results in this paper were derived on the basis of maximum likelihood estimation and Wald-type tests based on the sampling variance of the estimator. Results may differ if one is interested in properties of other tests, such as the power of the likelihood ratio test. For example, effective error of the slope component in a LGCM may then also depend on intercept variance and intercept-slope covariance (Brandmaier et al., 2018b).

### Outlook

PM designs for longitudinal data can also be extended to include multi-form designs on the item level (Rhemtulla et al., 2014). Then participants are not only a part of a partial *wave* pool but also part of partial *item* pools. That is, participants do not need to answer all items of a questionnaire at a particular occasion. Wave-level and item-level missing designs can be combined (Rhemtulla et al., 2016) and in principle be analyzed by the same logic proposed here using an effective error estimate for multiple indicator LGCMs (see von Oertzen et al., 2010). For example, following the logic presented earlier, we can derive the effective error of a longitudinal three-form missing design, in which participants in each block are randomly assigned to either the first half or the second half of items, which yields a design that minimizes both the number of assessments per person but also the number of items administered to each person.

In future work, effective error could be extended to account for auto-regressive processes (also see Willett, 1989). Generally, equal spacing of measurements may still be a viable option because it allows testing for other shapes of change. Again, we want to emphasize here that—if possible—researchers should explore the design space to the best possible extent instead of applying rules of thumb. We hope that the asymptotic approach presented here will support researchers in the quest for optimal designs to save resources, reduce study participants’ load, and optimize the statistical power of their study designs.

## Footnotes

1 Assuming that multiple independent measurements can happen at virtually the same time (practically, on the same day).

2 In particular, they showed that power remained identical when a complete wave was removed while the total study time (13 years) had to be extended by a mere 11 days. See Fig. 2 in von Oertzen and Brandmaier (2013) for an illustration of further possible trade-offs.
